# Comparison of the antioxidant effects of carnosic acid and synthetic antioxidants on tara seed oil

**DOI:** 10.1186/s13065-018-0387-4

**Published:** 2018-04-04

**Authors:** Zhan-jun Li, Feng-jian Yang, Lei Yang, Yuan-gang Zu

**Affiliations:** 1 Key Laboratory of Forest Plant Ecology, Ministry of Education, Northeast Forestry University, Harbin, 150040 China; 2 Yichun Academy of Forestry, Yichun, Heilongjiang Province, 153000 China

**Keywords:** Carnosic acid, Tara seed oil, Antioxidant, Oxidative stability

## Abstract

**Background:**

In the present study, tara seed oil was obtained by supercritical fluid extraction and used to investigate the antioxidant strength of carnosic acid (CA) compared with conventional synthetic antioxidants.

**Methods:**

The antioxidants were added to the tara seed oil at 0.2 mg of antioxidant per gram of oil. The samples were then submitted to at 60 °C 15 days for an accelerated oxidation process, with samples taken regularly for analysis. After oxidation, the samples were analyzed to determine the peroxide value, thiobarbituric acid reactive substances, conjugated diene content, and free fatty acid content. CA was investigated at three purity levels (CA20, CA60, CA99), and compared with three synthetic antioxidants (butylatedhydroxyanisole, butylatedhydroxytoluene, and tert-butylhydroquinone).

**Results:**

The oxidation indicators showed that CA was a strong antioxidant compared to the synthetic antioxidants. The antioxidant activities decreased in the order: tert-butylhydroquinone > CA99 > CA60 > CA20 > butylatedhydroxyanisole > butylatedhydroxytoluene. These results show that CA could be used to replace synthetic antioxidants in oil products, and should be safer for human consumption and the environment. 
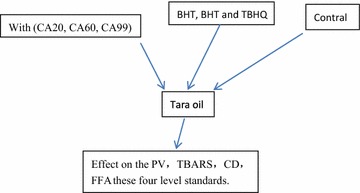

## Introduction

As an important plant tannis, tara (*Caesalpiniaspinosa*) is a kind of precious tree which represents significantly economic benefit, ecological benefit and social benefit. Besides, oil extracted from tara seeds has high content of unsaturated fatty acids. In recent years, it has received extensive attention among researchers [1]. The eight major fatty acids in tara seed oil are palmitic acid, palmitoleic acid, stearic acid, oleic acid, linoleic acid, arachidonic acid, linolenic acid, and behenic acid. The dominant unsaturated fatty acids are linoleic acid, oleic acid, behenic acid, and linolenic acid with contents of 65.36%, 13.33%, 2.30% and 0.99%, respectively [2, 3]. Its comprehensive exploitation and utilization is relatively low and correlation studiesand reports are rarely seen, so tara can be studied and developed deeply as an energy plant.

To date, some research has been conducted on tara seed oil and its applications, but this area of research is still in its infancy. The unsaturated double bonds in tara seed oil are sensitive, the unsaturated double bonds present in the fatty acids of tara oil are sensitive to oxidation, which may affect the overall quality of the oil [4, 5]. Exposure of tara seed oil to high temperatures and light can result in oxidation and increase the peroxide value (PV), which makes the oil unpalatable [6, 7]. The PV is an indicator of the peroxide content and degree of oxidization of an oil. It can be used to determine the degree of lipid oxidation and deterioration, and is mainly used to measure the formation of lipid oxidation products in initial stages of oxidation. It provides a measure of the degree of oil rancidity, and a higher PV is generally indicative of a higher the degree of rancidity. High temperatures and exposure to light are known to promote peroxide formation [8, 9]. The oil is then not beneficial for human consumption because of its rancidity, and increased content of free radicals that are produced by oxidation [10–13]. Tara seed oil with a higher content of unsaturated fatty acids, especially polyunsaturated fatty acids, is more susceptible to oxidation than oil with a lower content of unsaturated fatty acids [14]. Oxidation of lipids in oils can produce rancid odors, unpleasant flavors, and discoloration, and also decrease the nutritional quality and safety because the resulting degradation products can have harmful effects on human health [15, 16].

Oxidation can occur during oil storage and transportation, and the addition of appropriate antioxidants can inhibit free radical generation and stop rancidification [17]. Currently, the most commonly used type of antioxidants are synthetic ones such as (BHA), (BHT), and (TBHQ) [18]. Studies have shown that these synthetic antioxidants can have differing degrees of toxicity in humans, and can affect the liver, spleen, and lungs [19–21].

The antioxidant strength of a compound can be evaluated by investigating its effect on a number of oxidation indicators, including PV, thiobarbituric acid reactive substances (TBARS), conjugated diene (CD) content, and free fatty acid (FFA) content. In the present study, the antioxidant abilities of carnosic acid (CA) and the synthetic antioxidants BHA, BHT, and TBHQ in tara seed oil were compared. Carnosic acid is a phenolic (catecholic) diterpene, endowed with antioxidative and antimicrobial properties. These results provide a theoretical basis for application of CA to preservation of oils during storage and transportation.

## Materials and methods

### Materials

Refined, bleached, and deodorized tara seed oil was obtained by Supercritical Fluid Extraction from tara powder (60 mesh) prepared from fresh tara seeds (Wonderful variety) that were collected from Yunnan Province, China in September, 2014. The α-tocopherolactalso also as an antioxidant, which content was very low (< 4.3 mg kg−1), and the oil contained no synthetic antioxidants, all reagents and solvents were either of HPLC or analytical grade. BHA, BHT, TBHQ, Folin–Ciocalteu reagent, gallic acid standard, catechin standard, and free radicals, and CA were purchased from Sigma-Aldrich Co. (St. Louis, MO, USA).

### Preparation of oil

Fresh tara seeds were dried in an oven at 45 °C to constant mass, and then ground into powder (60 mesh). Oil was extracted from the powder using supercritical fluid extraction under the following conditions: extraction time 120 min, extraction temperature 45 °C, and extraction pressure 35 MPa, CO_2_ was the only fluid used.

110 mL of tara oil were placed in 125-mL brown-colored reagent bottles with narrow necks. The bottles were divided into seven groups, with each group containing three bottles for replication of the experiments. One group of bottles was designated as the blank controls, and CA of different purity (CA20, CA60, and CA99) was added to the first group experiment with three differents bottles at 0.2 mg of CA per gram of oil. The other six groups experiments were designated as synthetic antioxidant groups, and BHA, BHT, TBHQ were added at 0.2 mg of antioxidant per gram of oil [22, 23]. Each bottle was placed on a magnetic stirrer for 30 min to thoroughly mix the antioxidant and oil. The bottles were then placed in an incubator at 60 °C for 15 d to induce accelerated oxidation. 2 ml aliquots were taken from each bottle every 3 days. The samples were analyzed for the PV, TBARS, CD and FFA to determine the effects of different types of antioxidants on the oxidation stability of tara seed oil [24, 25].

### PV

The PV were measured according to the method of the AOAC [26], with slight modifications. Accordingly, the tara seed oil samples (2 g) were dissolved in 30 mL of a chloroform-glacial acetic acid (3:2, v/v) solution. Then, 1 mL of a saturated solution of KI was added. The mixture was shaken by hand for 1 min and then kept in dark for 5 min. After the addition of 75 mL of distilled water, the mixture was titrated against sodium thiosulfate (0.002 mol/L) until the yellow color almost disappeared. Then, 0.5 mL of starch indicator solution was added. Titration was continued until the blue color disappeared. The blank was treated exactly like the samples. The PV (milliequivalents (meq) of peroxide per kilogram of oil, meq/kg) was calculated as follows:

$${\text{PV }}\left( {{\text{meq}}/{\text{kg}}} \right) \, = { 12}. 6 9 { } \times { 78}. 8 { } \times {\text{ C }}\left( {{\text{V1 }}{-}{\text{ V}}0} \right) \, /{\text{m}},$$

where C is the concentration of sodium thiosulfate (mol/L); V1 and V0 are the volumes (mL) of sodium thiosulfate used in the sample and blank titrations, respectively; and m is the mass (g) of tara seed oil.

### TBARS

TBARS is defined as the quantity of malondialdehyde (in milligrams) present in 1 kg of sample, and is an index of lipid oxidation as measured by MDA content. TBARS were determined using a slight modification of the method by Zhang et al. [27]. Tara seed oil samples (2 g) were homogenized in 10 mL of a trichloroacetic acid (7.5%) and EDTA (0.1%) aqueous solution. The samples were shaken continuously for 30 min on a mechanical shaker and then filtered. Exactly 5 mL of the filtrate was added to 5 mL of 2-thiobarbituric acid (2.88 g/L) solution, and then transferred to a 25-mL colorimetric tube. The mixture was heated in a water bath at 90 °C for 40 min until a pink color developed. Then, the tube was cooled for 1 h, and centrifuged for 5 min (room temperature). The supernatant was added to 5 mL of chloroform in another tube and then shaken. The mixture was left to stand for at least 1 h, and then the absorbance was measured at 532 nm using a spectrophotometer (UV-2550, Shimadzu, City, Country). The TBARS content was calculated from a malondialdehyde (MDA) standard curve. The MDA solutions were freshly prepared by acidification of 1,1,3,3-tetraethoxypropane. The standard curve covered a concentration range of 0.02–0.3 µg/mL, the results are expressed as milligrams of MDA per kilogram of the tara seed oil. The MDA concentration was calculated as follows:

$${\text{MDA }}\left( {{\text{mg}}/{\text{kg}}} \right) = {\text{ S/m }} \times {1}0,$$

where S is the mass concentration of MDA obtained from the standard curve, and m is the mass of squalene (µg) in the sample.

### CD

The CD content was measured using a slight modification of the method proposed by Leclerc et al. [28]. Oil samples (0.02 g) were diluted with isooctane, and the absorbance of each solution at 233 nm was determined against a blank of isooctane. The CD content was calculated from the absorbance and the final concentration of the sample as follows:

$${\text{CD }} = {\text{ A}}/{\text{C}} \times {\text{P}},$$

where A is the absorbance of the sample at 233 nm; C is the final concentration of the sample after dilution (grams per 100 mL of isooctane); and P is the path length of the cell (cm).

### FFA

FFA determinations were performed according to the method of Zhang et al. [29], with some modifications. Oil samples (3 g) were dissolved in 50 mL of a mixture of neutral ether–ethanol (1:1, v/v). The mixture was then shaken by hand. After cooling to room temperature, the mixture was titrated against potassium hydroxide (0.01 mol/L) using phenolphthalein (10 g/L) as an indicator. The FFA value (meq/kg) was calculated as follows:

$${\text{FFA }}\left( {{\text{mg}}/{\text{g}}} \right) = \left( {{\text{V }} \times {\text{ C }} \times {56}.11}\right){\text{/m}},$$

where V is the volume of potassium hydroxide used in the titration with the samples (mL); C is the concentration of potassium hydroxide (mol/L); and m is the mass of the tara seed oil (g) sample.

### Statistical analysis

Statistical analyses (ANOVA) were performed using SPSS11.5 (Company name, City, Country). The results are expressed as the mean standard deviation. Results with P < 0.05 were considered significant. Each group experiment was repeated three times, and take the results were averaged results as the final experimental data.

## Results and discussion

### Effect of CA on the PV

**Fig. 1:**
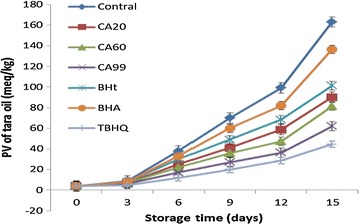
PV results for tara seed oil samples after accelerated oxidation

Figure 1 shows the PV results for the tara seed oil samples with added CA20, CA60, CA99, BHA, BHT, and TBHQ were obtained after accelerated oxidation at 60 °C (Fig. 1).

The PV for each tara seed oil increased as the length of storage increased, which resulted in production of more primary oxidation products (e.g. hydroperoxides). For the experimental control group without any antioxidant, the PV increased faster and reached a higher value (163.1 ± 0.35 meq/kg) than the samples with antioxidants. Addition of one of the antioxidants (CA20, CA60, CA99, BHA, BHT, or TBHQ) decreased the PV. The PVs for CA20, CA60, CA99, BHA, BHT, and TBHQ were 90.1 ± 0.61 meq/kg, 81.4 ± 0.42 meq/kg, 62.0 ± 0.31 meq/kg, 136.5 ± 0.55 meq/kg, 101.3 ± 0.46 meq/kg, and (44.6 ± 0.49) meq/kg, respectively. Compared with the control group, the PV inhibition rates of the antioxidants CA20, CA60, CA99, BHA, BHT, and TBHQ were 44.8, 50.1, 62.0, 16.3, 37.9, and 72.7%.

These results show that CA effectively inhibited oxidation of tara seed oil, and was a stronger antioxidant than BHA and BHT but a weaker antioxidant than TBHQ. The antioxidants could be arranged in order of antioxidant strength as follows: TBHQ > CA99 > CA60 > CA20 > BHA > BHT.

### Effect of CA on TBARS

Lipid oxidation generates primary oxidation products, which reduce the stability of the product and can result in further oxidation and decomposition. Further oxidation generates secondary oxidation products, such as ketones, aldehydes, and acids. Among these secondary oxidation products is MDA, which can be detected by measuring the absorbance at 532 nm MDA can be generated during oil oxidation, and can be used as an indicator of rancidity.

**Fig. 2:**
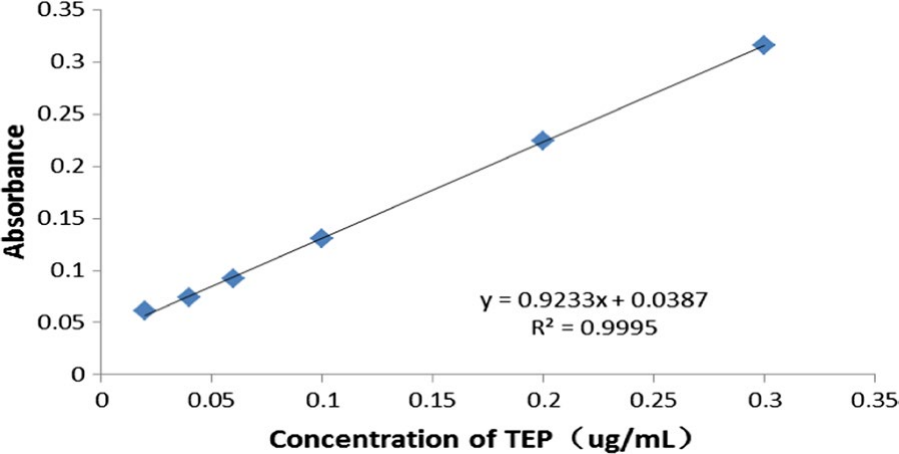
MDA standard curve

The standard curve of MDA (Fig. 2) gave an equation of ${\text{y}} = 0. 9 2 3 3 {\text{x}} + 0.0 3 8 7 { }({\text{R2}} = 0. 9 9 9 5)$.

**Fig. 3:**
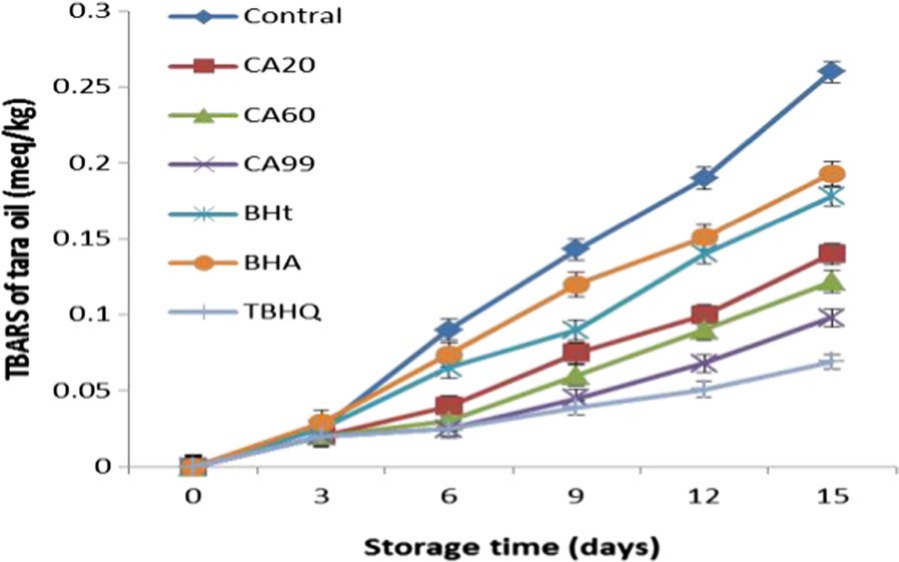
TBARS results for tara seed oil samples after accelerated oxidation

TBARS results were obtained for tara seed oil with the six antioxidants (Fig. 3).

Compared with the control group without antioxidant (TBARS = 0.26e of 1 meq/kg), all the antioxidants reduced the TBARS. The TBARS results for CA20, CA60, CA99, BHA, BHT, and TBHQ were 0.14 ± 0.004 meq/kg, 0.122 ± 0.005 meq/kg, 0.098 ± 0.003 meq/kg, 0.193 ± 0.006 meq/kg, 0.178 ± 0.005 meq/kg, and 0.069 ± 0.001 meq/kg, respectively. The TBARS inhibition rates for CA20, CA60, CA99, BHA, BHT, and TBHQ were 46.2, 53.1, 62.3, 25.8, 31.5, and 73.5%, respectively.

These results show that CA is an effective antioxidant for reducing oxidation of tara seed oil. Compared with the other antioxidants, CA was stronger than BHA and BHT but weaker than TBHQ. The antioxidants could be arranged in order of antioxidant strength as follows: TBHQ > CA99 > CA60 > CA20 > BHA > BHT.

### Effect of CA on CD

The CD content is frequently used as an indicator of hydroperoxide content, as proposed by Lecomte J et al. Most hydroperoxides formed through oxidation of unsaturated fatty acids are conjugated dienes. Formation of hydroperoxides stabilizes the radical state through formation of the double bond, which absorbs in the UV region (235 nm). The CD content is an indicator of the oxidative state of an oil, and of the effectiveness of an antioxidant.

**Fig. 4:**
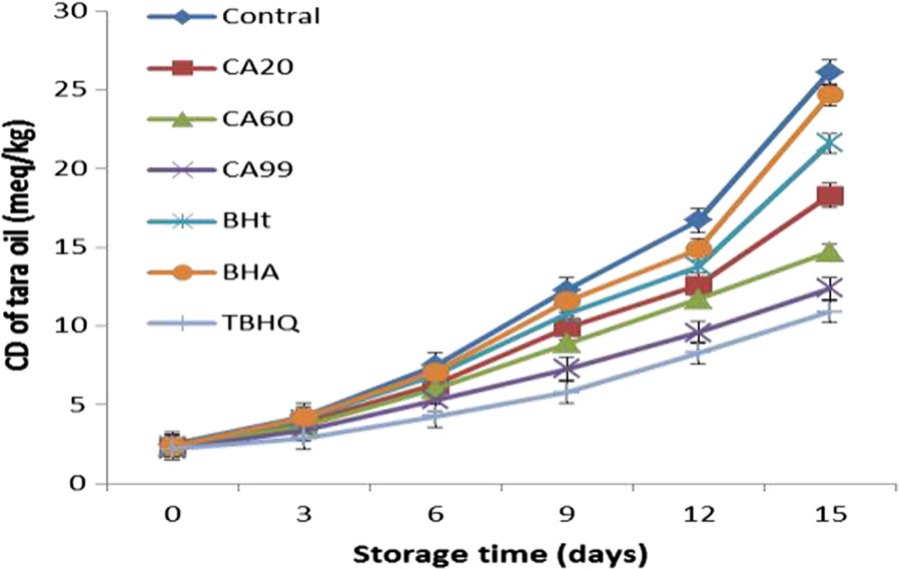
CD results for tara seed oil samples after accelerated oxidation

CD results were obtained for tara seed oil CD (Fig. 4).

Compared with the control group, all the antioxidants improved the stability of tara seed oil to oxidation. The control group had a CD value of 26.1 ± 0.02 at 15 days. The CD values at 15 days for the samples with CA20, CA60, CA99, BHA, BHT, and TBHQ were 18.3 ± 0.01, 14.7 ± 0.005, 12.4 ± 0.021, 24.7 ± 0.015, 21.6 ± 0.02, and 10.9 ± 0.017, respectively. The inhibition rates for CD content for CA20, CA60, CA99, BHA, BHT, and TBHQ were 29.9, 43.7, 52.5, 5.4, 17.2, and 58.2%, respectively.

These results show that CA is a good antioxidant for tara seed oil. Compared with the other antioxidants, CA is stronger than BHA and BHT, and weaker than TBHQ. The antioxidants could be arranged in order of antioxidant strength as follows: TBHQ > CA99 > CA60 > CA20 > BHA > BHT.

### Effect of CA on FFA

Temperature, light, and other factors can cause oil oxidation, which generates both primary and secondary oxidation products. During oil degradation, triglyceride hydrolysis, which forms FFAs, and fatty acid dissociation occur. The FFA content can be used to determine the degree of oil oxidation.

**Fig. 5:**
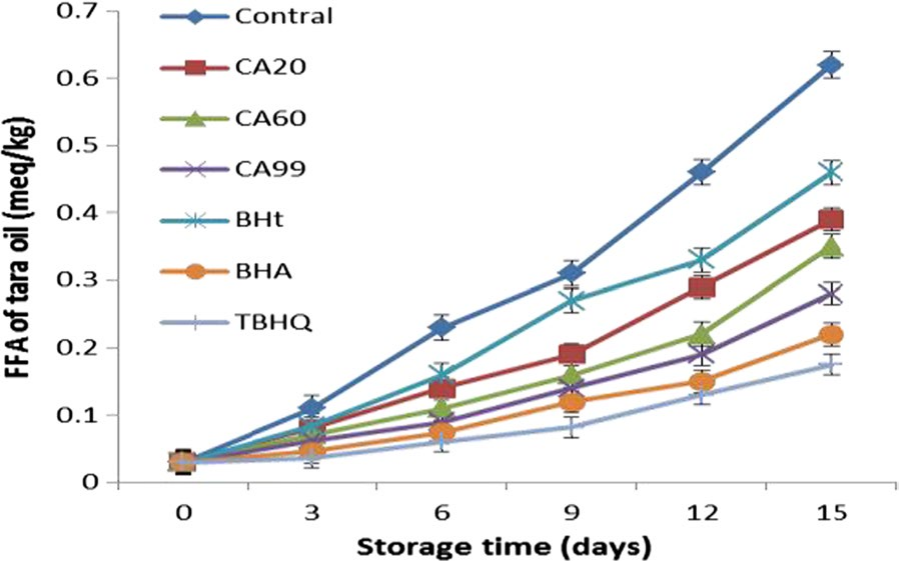
FFA results for tara seed oil samples after accelerated oxidation

FFA results were obtained for tara seed oil (Fig. 5).

Compared with the control group without antioxidant (15-day FFA = 0.62 ± 0.04%), all of the antioxidants decreased the FFA content. For CA20, CA60, CA99, BHA, BHT, and TBHQ the 15-day FFA results were 0.39 ± 0.04%, 0.35 ± 0.354%, 0.28 ± 0.284%, 0.22 ± 0.224%, 0.46 ± 0.464%, and 0.175 ± 0.05%, respectively. The FFA inhibition rates of CA20, CA60, CA99, BHA, BHT, and TBHQ were 37.1, 44.0, 55.0, 65.0, 26.0, and 72.0%, respectively.

These results show that CA is a good antioxidant for tara seed oil. Compared with the other antioxidants, CA is stronger than BHA and BHT, and weaker than TBHQ. The antioxidants can be arranged in order of antioxidant strength as follows: TBHQ > CA99 > CA60 > CA20 > BHA > BHT.

## Conclusions

The antioxidant strength of CA was compared with synthetic antioxidants by adding CA20, CA60, CA99, BHA, BHT, and TBHQ to tara seed oil and exposing the samples to accelerated oxidation conditions. Analysis of oxidation indicators, including PV, TBARS, CD content, and FFA content, was used to determine the effect of each antioxidant. The results on the last day of accelerated oxidation (day 15) were compared, and showed that CA was a stronger antioxidant than the synthetic antioxidants. In order of antioxidant strength, the antioxidants were TBHQ > CA99 > CA60 > CA20 > BHA > BHT. Therefore, CA could be used to replace synthetic antioxidants, and will likely be safer for human consumption and the environment because of its lower toxicity.
